# Supplementary material for: Gain-of-function mutation in SKAP2 leads to type 1 diabetes and broader autoimmunity through hyperactive integrin signaling in myeloid cells
Source: bioRxiv. 2026 Apr 6:2026.04.02.716136. Preprint. [Version 1] doi: 10.64898/2026.04.02.716136 (PMC13081829; doi:10.64898/2026.04.02.716136)
Supplement: Supplement 1 [file media-1.pdf]

Supplemental Data

FIGURE S1

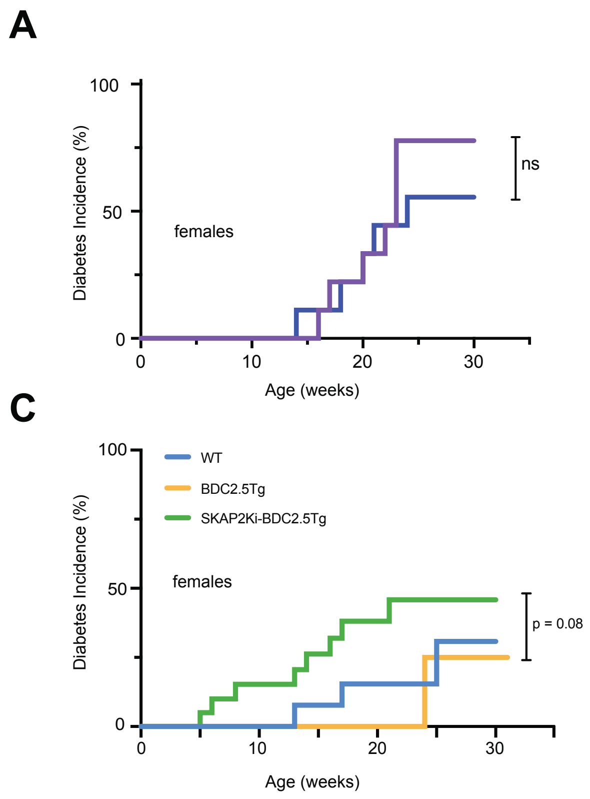

Supplemental Figure 1. **The SKAP2 G153R mutation accelerates autoimmune diabetes and breaks tolerance in the BDC2.5 transgenic model.**

(A, B) Cohorts of WT and SKAP2$^{G153R/+}$ female **(A)** and male **(B)** mice were monitored for blood glucose levels weekly. Diabetes incidence (blood glucose > 200 mg/dl on two tests separated by one week) incidence is shown onset and incidence over 30 – 40 weeks is shown (females: WT, n=9; SKAP2$^{G153R/+}$, n=10; males: WT, n=10; SKAP2$^{G153R/+}$, n=10). **(C)** Cohorts of female WT, BDC2.5 transgenic mice (on the NOD genetic background) and SKAP2$^{G153/+}$ - BDC2.5 transgenic mice were monitored for blood glucose levels weekly as above. Cohort sizes: WT, n=15; BDC2.5 Tg, n=10; SKAP2$^{G153R/+}$- BDC2.5, n=20). Diabetes onset curves were analyzed using Log-rank testing (Mantel-Cox test); *=p<0.05

FIGURE S2

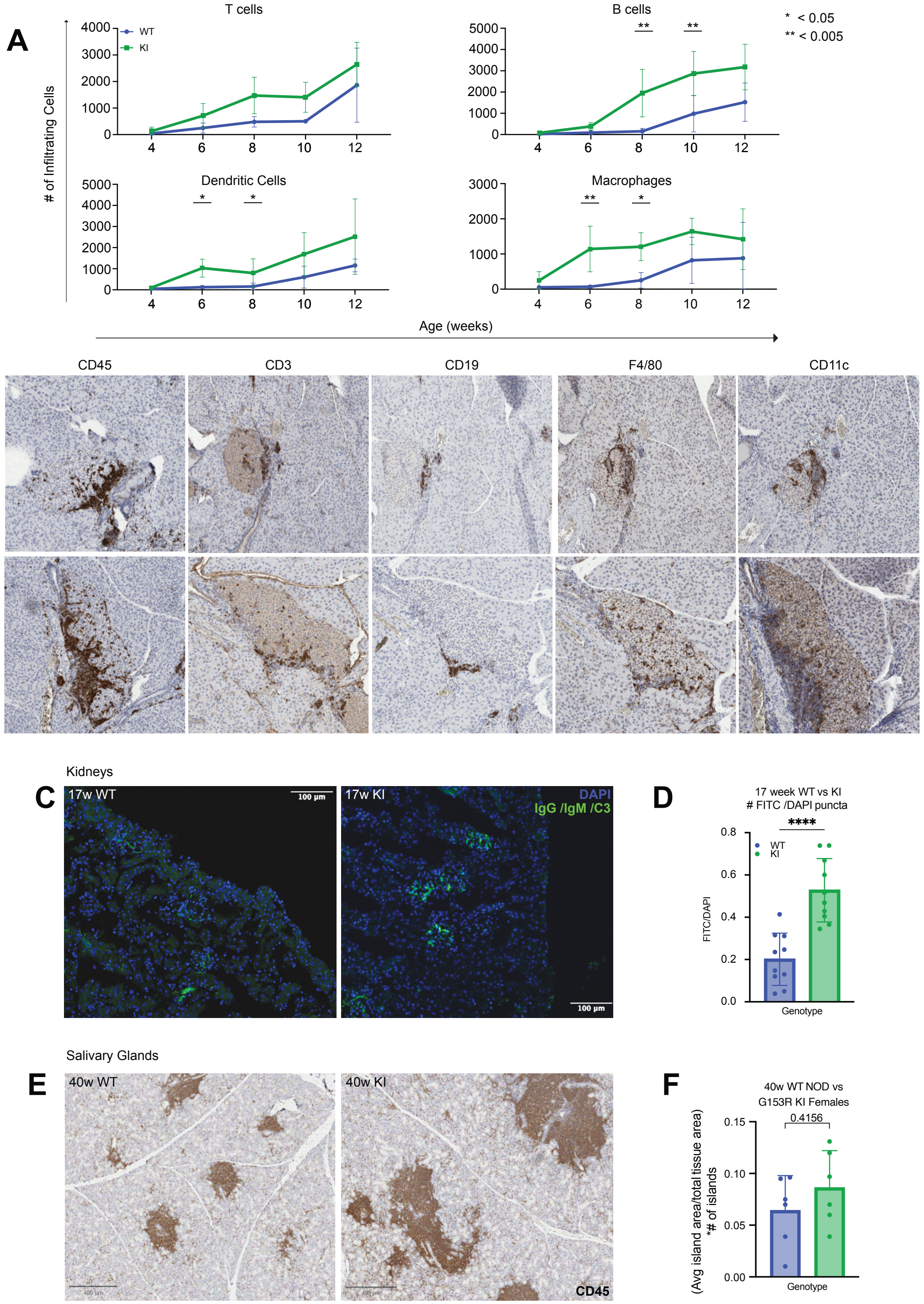
A
T cells
B cells
Dendritic Cells
Macrophages
WT
KI
* < 0.05
** < 0.005
# of Infiltrating Cells
Age (weeks)
B
CD45
CD3
CD19
F4/80
CD11c
6w WT
6w KI
Kidneys
C
17w WT
17w KI
DAPI
IgG /IgM /C3
100 μm
D
17 week WT vs KI
FITC /DAPI puncta
****
FITC/DAPI
Genotype
Salivary Glands
E
40w WT
40w KI
CD45
F
40w WT NOD vs
G153R KI Females
0.4156
(Avg island area/total tissue area)
*# of islands
Genotype

Supplemental Figure 2. **Histologic analysis of NOD WT vs SKAP2 KI mice.**

(A) Pancreases from female WT and SKAP2$^{G153R/G153R}$ (KI) mice were prepared for histology as described in the methods – staining for CD45, CD3, CD19, F4/80, and CD11c (see Table S4 for antibodies used) to detect total leukocytes, T cells, B cells, macrophages, and dendritic cells, respectively. Pancreases from mice of the indicated age (4, 6, 8, 10, 12) were used. Scanned images of 10 different islets were analyzed for leukocyte infiltration by QuPath software to quantify cell numbers. Each age cohort had n=5 mice each. Data for each cell type at each time point were averaged and plotted as individual line graphs. P values were calculated by two-way ANOVA. *=p<0.05; **=p<0.01. **(B)** Representative images of pancreatic islets, stained for the above markers, from 6-week-old female WT and KI mice. **(C–D)** Immune complex deposition in the kidneys of 17-week-old WT and KI mice was assessed by IgG/IgM/C3 immunofluorescence staining and quantification. **(E–F)** CD45^{+} immune infiltration in salivary glands of 40-week-old WT and KI mice and corresponding quantification. In bar graphs, each dot represents samples from a single mouse. P values were calculated by unpaired t-tests. Data represent the difference between means ± SEM; ****=p<0.0001.

FIGURE S3

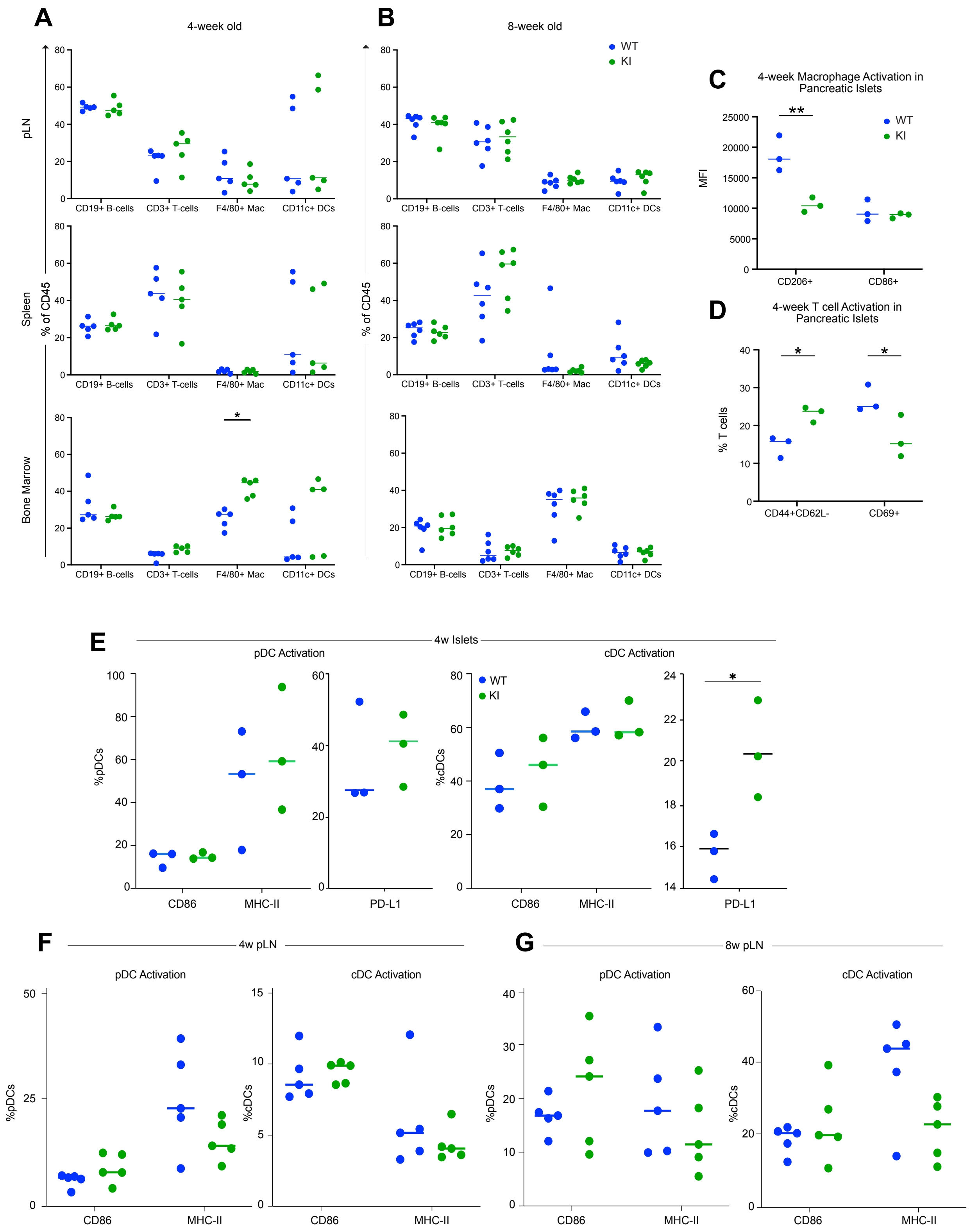

A
4-week old
B
8-week old
WT
KI
pLN
Spleen
Bone Marrow
% of CD45
CD19+ B-cells
CD3+ T-cells
F4/80+ Mac
CD11c+ DCs
C
4-week Macrophage Activation in Pancreatic Islets
MFI
CD206+
CD86+
D
4-week T cell Activation in Pancreatic Islets
% T cells
CD44+CD62L-
CD69+
E
4w Islets
pDC Activation
cDC Activation
%pDCs
%cDCs
CD86
MHC-II
PD-L1
F
4w pLN
G
8w pLN

Supplemental Figure 3. **Flow cytometric analysis of WT vs SKAP2 KI mice.**

Multiparameter flow cytometry was conducted on pancreatic draining lymph node (pLN), spleen, and bone marrow tissues isolated from four or eight-week-old female WT versus SKAP2$^{G153R/G153R}$ (KI) mice. Cell suspensions from the tissues were stained with antibodies listed in Table S4, then analyzed on an Aurora Spectral Flow cytometer. **(A, B)** The percentages of B cells, T cells, macrophages, and dendritic cells within each tissue are shown (n=5 mice per group – each dot represents one animal). **(C)** The mean fluorescence intensity (MFI) of the marker CD206 or CD86 on F4/80^{+} macrophages from isolated pancreatic islets of four-week-old mice is shown on the upper panel. **(D)** The percentages of activated (CD44^{+}CD62L^{-}) or replicating (CD69^{+}) T cells from the same islet samples are shown. **(E)** Markers of DC activation (MHC II, CD86, and PD-L1) were stained on islet cell preparations, and the percentages of CD11c^{+} pDCs or cDCs displaying these markers are shown in samples from four-week-old NOD WT and SKAP2$^{G153R/G153R}$ (KI) mice. **(F)** Markers of DC activation in cDCs and pDCs from pancreatic draining lymph nodes (LN) are shown. Cells from Islets and draining LN were pooled from n=3 mice per group for cell suspension generation. Note that enumerating the total number of immune cells within the isolated islets by flow cytometry was not useful, as many immune cells remained within the pancreatic tissue after islet removal. Histology proved more reliable for total cell counting. P values were calculated using two-way ANOVA analysis; *=p<0.05; **=p<0.01.

FIGURE S4

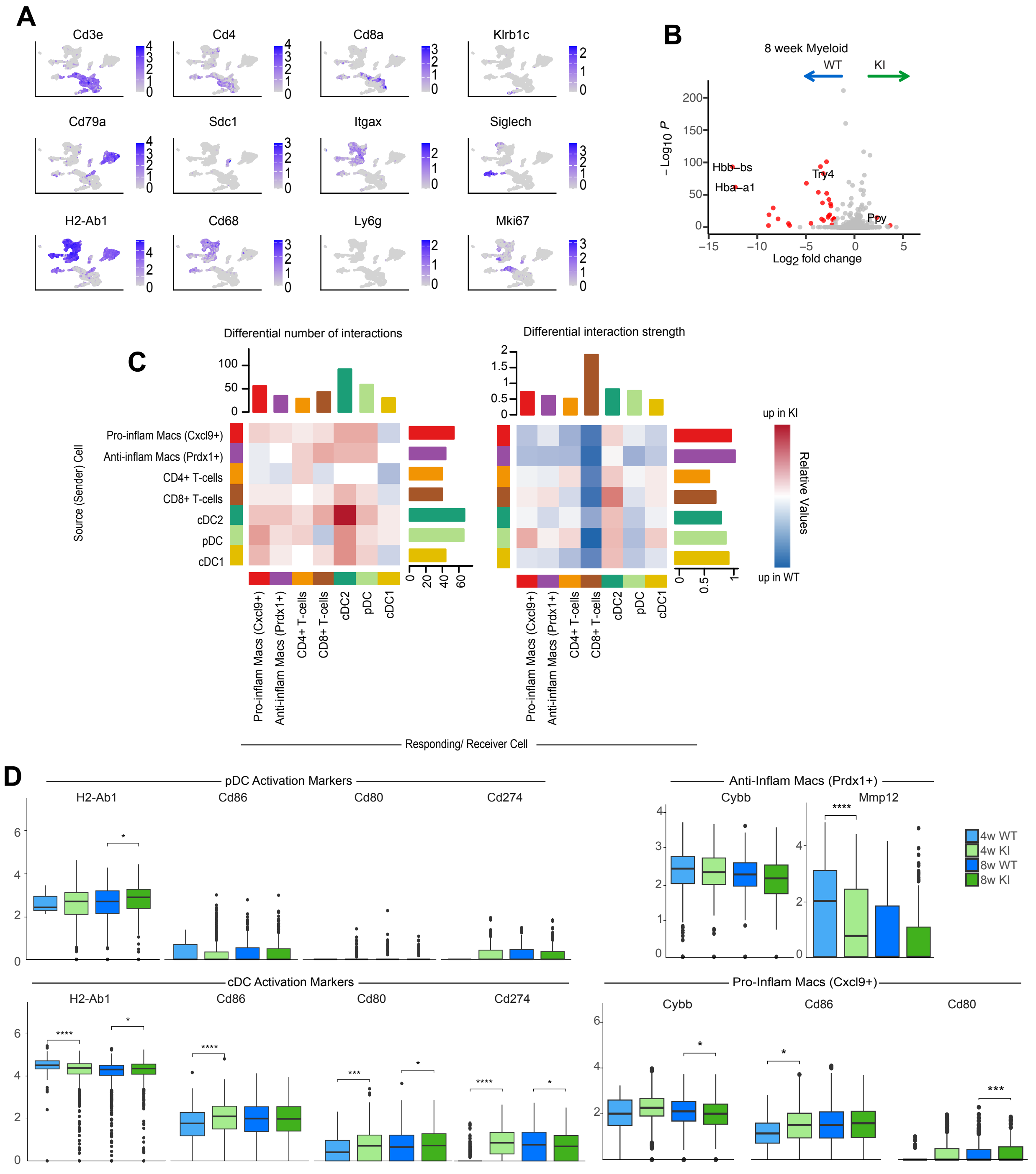

Supplemental Figure 4. **Gene set, Cell Chat, and individual transcript analysis of immune cells from islets of WT versus SKAP2$^{G153R/G153R}$ KI mice.**

(A) Individual feature plots for specific lineage-defining transcripts (shown on the overall plot of islet cells from four-week-old SKAP2$^{G153R/G153R}$ mice). (**B)** Gene set analysis (GSEA) of all CD45^{+} cells from eight-week-old WT versus SKAP2$^{G153R/G153R}$ (KI) mice, with differences plotted in a volcano plot. Red dots indicate genes showing $\log_2$ fold difference > 0.3 and $-\log_{10}$ > 1.3 (giving a $p<0.05$), which are default cutoffs in Seurat/Volcano packages. This analysis indicates that by eight weeks of age, few differences in leukocyte gene expression patterns were observed between WT and KI mice. **(C)** Cell Chat data plotted as a box plot, showing the number of predicted cellular interactions and interaction strength between islet immune cells in WT and SKAP2$^{G153R/G153R}$ (KI) mice. Differential interactions are represented by red versus blue boxes in the two plots. The relative increase in red boxes on the left suggests that the KI immune cells will form more interactions, while the blue boxes on the right indicate that the WT cells will exhibit stronger interactions. **(D)** Transcript read counts for various activation makers and inflammatory proteins in sorted DC and macrophage subsets. Shown are counts for the transcripts for encoding MHC II, CD80, CD86, and CD274 in DC subsets (cDCs versus pDCs), along with *Cybb* (encoding NOX2) and Mmp12 in macrophage subsets. Each dot represents the read count for the indicated gene in a single cell. P values were calculated using two-way ANOVA analysis; *=p<0.05; **=p<0.01, ***=p<0.001, ****=p<0.0001.

FIGURE S5

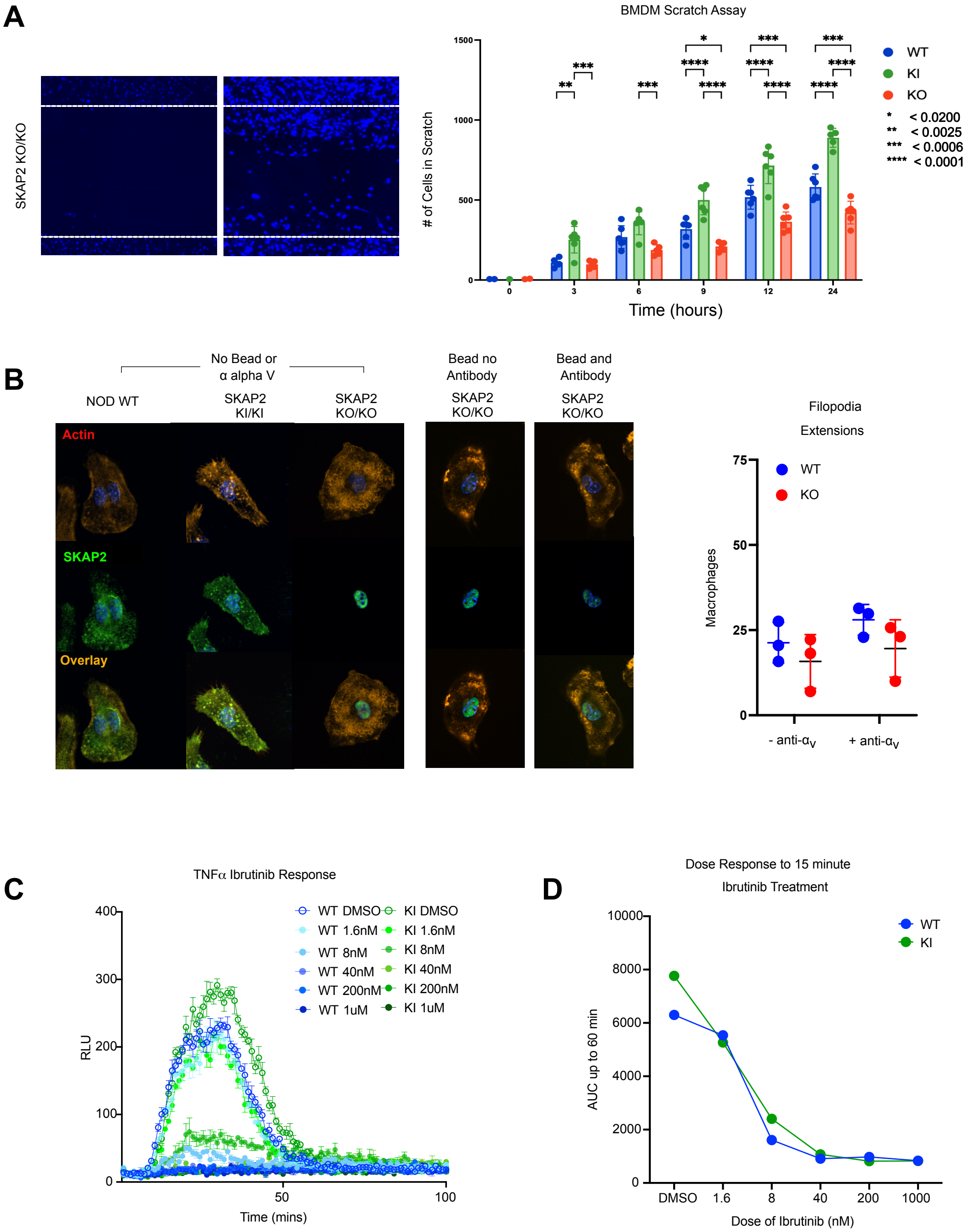

Supplemental Figure 5**. Comparison of integrin signaling in WT versus SKAP2 G153R versus *Skap2*$^{-/-}$ macrophages and neutrophils.**

(A) Bone marrow-derived macrophages (BMDM) from female WT, homozygous *Skap2* G153R (KI), and S*kap2*$^{-/-}$ (KO) mice were expanded on tissue culture plates, and scratch assays were performed as described in the methods. The left panel shows migration responses in *Skap2*$^{-/-}$ cells, compared with the micrographs in Fig. 6a. The right panel shows a time course of WT, KI, and KO cell migration into scratched areas. Each dot represents a separate scratch culture. Cell counting was performed using QuPath software. The statistical significance of differences between groups is shown. **(B)** Confocal micrographs of WT, KI, or KO BMDMs plated on glass coverslips, then stimulated with uncoated polystyrene beads or with beads coated with anti-αv integrin antibody for 20 min, then fixed and stained for F-actin with phalloidin (red) and anti-Skap2 (green). Merged images show the overlap of the stains in yellow. Filopodia extensions were quantified in unstimulated versus stimulated WT and KO cells. **(C)** Bone marrow-derived neutrophils from WT or KI mice were pre-treated with the indicated doses of ibrutinib for 15 min, then plated on poly-RGD-coated wells with TNFα. ROS production was monitored by isoluminol reduction as described in the methods. **(D)** The total area under each curve (from 0 to 60 min) in panel c is shown for each ibrutinib dose. These data indicate that WT and KI neutrophils show the same dose-response to ibrutinib inhibition of integrin signaling in this ROS production assay.

FIGURE S6

A

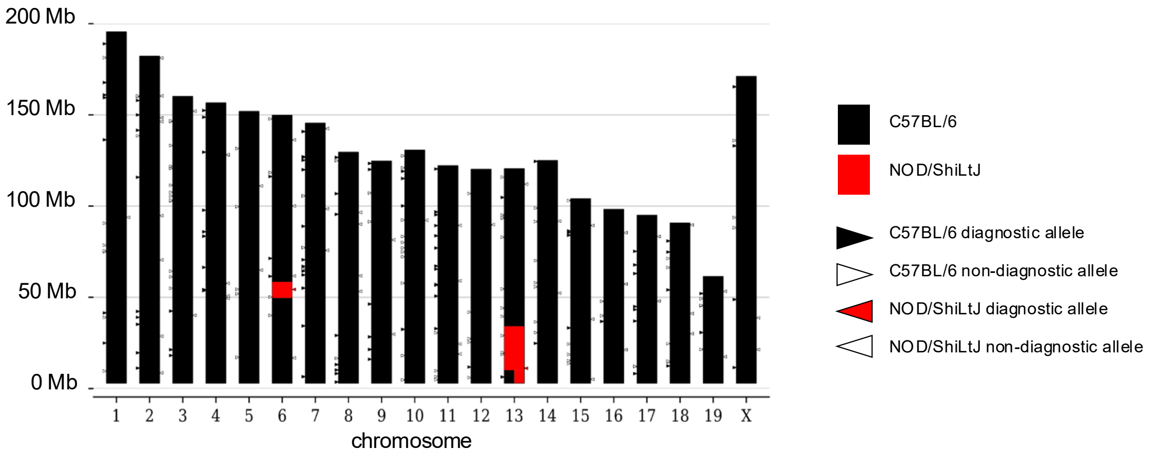

B

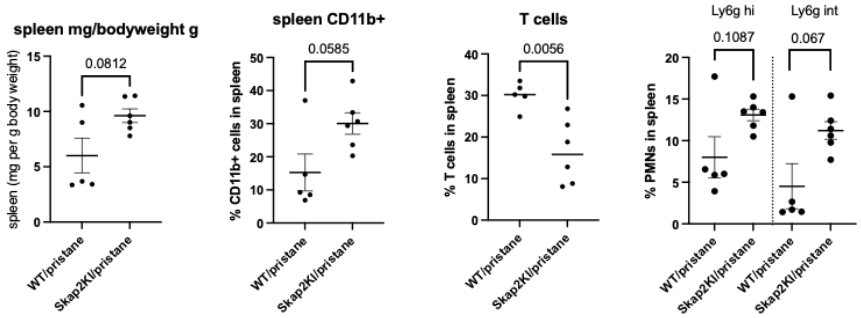

C

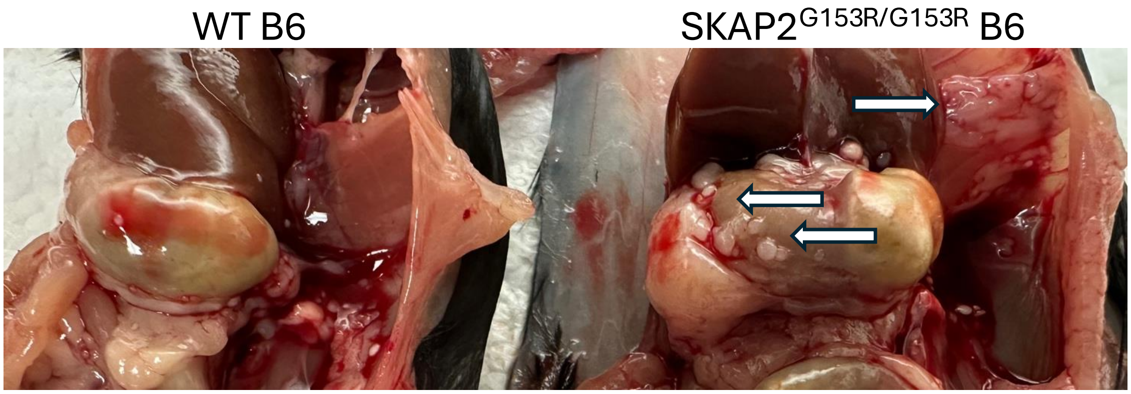

Supplemental Figure 6. **Pristane induced inflammation in WT B6 versus SKAP2$^{G153R/G153R}$ B6 mice.**

(A) SKAP2$^{G153R/G153R}$ NOD mice were backcrossed to C57BL/6 for 12 generations, with the mutation confirmed by genotyping. The genetic background was analyzed by SNP genotyping of tail genomic DNA from breeders. Six breeders were tested and found to have an average of 5%

NOD and 95% C57BL/6 background strains. Summary data from one of the breeders is shown above. Mouse *Skap2* is found on chromosome 6 at ~51.8Mbp, and all mice showed NOD diagnostic markers in this region. Other regions of NOD were found on chromosome 8 (3/6 mice) and 13 (6/6 mice). **(B)** Spleen weights and cell counts in WT B6 versus SKAP2$^{G153R/G153R}$ mice at three months following pristane injection. P values were calculated using two-way ANOVA analysis. **(C)** Photomicrograph of peritoneal lymphoid aggregates (shown by white arrows) in WT B6 versus SKAP2$^{G153R/G153R}$ mice at three months following pristane injection.

Supplemental Table 1: List of antigens on array heat maps, from top to bottom, on Fig 2C:

AChR3	ErbB3&B4	RSVP antigen
AFP	ERP29	Rubella virus grade III antigen
ALDOC	Ferritin	Rubella virus grade IV antigen
Aldolase (muscle)	Flagellin CBir1	Rubeola antigen
Alpha-2-macroglobulin-like 1	FUCA1	Saccharomyces Cerevisiae
alpha-fodrin	Galactocerebroside	Salivary Gland Protein 1 (SP1)
Angiotensin II type 1 Receptor	GBM	SCCA
ANXA1	Glutamate decarboxylase-65/GAD2	SOX2
Apolipoprotein B/E	HEPATITIS A ANTIGEN	Sphingomyelin
Azurocidin	HPV E6 (16)	Tetanus toxoid
BAFF	HPV E7 (16)	TGF-beta1
beta-glucuronidase	HPV E7 (18)	Thrombopoietin (TPO)
BRCA1	HSV-1 ANTIGEN	Thyroglobulin
BRCA2	HSV-2 ANTIGEN	Tissue Transglutaminase (TTG)
C-MYC	IFN-alpha2	TNF-alpha
CA15-3	IFN-beta	TNF-beta
CA19-9	IFN-gamma	Toxoplasma
CA27-29	IL-1alpha	Troponin I
CA50	IL-1beta	Troponin I-T-C ternary complex mixture
CA72-4	IL-2	TWEAK (CD255)
CA125	IL-6	uPA
CA242	IL-8	VEGF-165
CAGE	IL-12 (p70)	VZV antigen
Calmodulin (brain)	IL-17A	VZV grade II antigen
Calreticulin(rabit calreticulin)	lactoferrin	
Carbonic Anhydrase 6 (CA6)	LC1	
Catalase	Lysosomal Associated Protein 2 (LAMP-2)	
Cathepsin G	MAG	
CCL2	MAGEA3	
CCL3	MBP	
CCL5	MMP-2	
CCL11	NSE	
CDK2	Nucleosome	
CEA	NY-ESO-1	
Cholinergic Receptor Muscarinic 3 (CHRM3)	OmpC	
CMV-G	Ox40L	
CMV-M	P53	
CMV EXT	Parotid Secretory Protein (PSP)	
CMV GRADE III ANTIGEN	PD1	
Complement Factor Hq	PD-L1	
CTLA4	Peptidyl Arginine Deiminases 1&2&3&4	
CXCL10	Phosphatidyl-l-serine	
Cytokeratin 19 Ag	PKM2	
dsDNA	Prostate Specific Antigen (PSA)	
EBV EBNA1	Prostate Specific Membrane Antigen (PSMA)	
EGF Receptor	Prostatic Acid Phosphatase	
Elastase	PSMA	
Endothelial Cell Extract	rhHSPG2	
Enolase	Rotavirus SA-11	
ErbB2	RSV antigen	

Supplemental Table 2: List of genes on PhIP-seq heat maps, from top to bottom, on Fig 2F:

4931409K22Rik
Actr10
Apc
Blnk
Bnc1
Cdc37
Cftr
Chd4
Cherp
Col11a2
Cp
D6Ertd527e
Dlx5
Fbxo28
Fhad1
Fhod3
Gm33989
Kirrel
Kmt2d
Lhx3
Mast2
Mpdz
Mre11a
Myrf
Nrg2
Olfr1129
Osbpl6
Ppfibp2
Prkab1
Psmb11
Ralgapa1
Reep6
Serpina3f
Shank3
Slf2
Snrpb
Snrpn
Spag8
Speg
Tex22
Trerf1
Uba1
Vldlr
Zfp207
Zfp78
Zfp846
Zmym4

Supplemental Table 3: List of genes on PhIP-seq heat maps, from top to bottom, on Fig. 7A:

4930452B06Rik	Tenm4
Abca4	Tet3
Arid1b	Trip10
Asxl3	Ttc23
Atad2b	Vwc2l
Atp10b	Vwc2l
Atp2b3	Wnt7b
Bpifb4	Zcchc2
C1qa	
Carmil1	
Cbarp	
Cntn5	
Crk	
Ddi2	
Eppk1	
Fam120c	
Fnbp1	
Gabra1	
Gm46637	
Golga4	
Grhl2	
Grhl3	
Hnrnpa1l2-ps2	
Hormad2	
Ikbip	
Kcnb1	
Lrrfip1	
Man1a	
Mcc	
Mrap2	
Mre11a	
Nhs	
Nom1	
Numa1	
Pdgfb	
Plch2	
Plec	
Pop1	
Ppan	
Prex1	
Ptges3	
Rad51b	
Rictor	
Rptor	
Rtf1	
Setd2	
Sned1	
Srcin1	
Tenm4	
Tet3	
Trip10	
Ttc23	
Vwc2l	
Wnt7b	
Zcchc2	
Srcin1	

Supplemental Table 4: Antibody staining reagents used:

Immunohistochemistry antibodies

Marker	Clone	Vendor	Dilution from stock
CD45	EPR20033	Abcam	1:1000
CD3	EPR22667-12	Abcam	1:500
CD19	EPR23174-145	Abcam	1:500
F4/80	D2S9R	Cell Signaling	1:300
CD11c	D1V9Y	Cell Signaling	1:300

MIBI reagents

Isotope	Metal	Antibody	Vendor	Clone	Micrograms/10^6 cells
31	P	Phosphorous (P)			1
39	K	K			1
40	Ca	Ca			1
56	Fe	Iron (Fe)			1
89	Y	Histone H3	CST	DIH2	0.25
141	Pr	FoxP3	Abcam	Polyclonal	5
143	Nd	CD4	Abcam	EPR19514	1.2
144	Nd	Cd11c	CST	D1V9Y	1.2
150	Nd	Granzyme B	Abcam	D6E9W	1.2
152	Sm	SKAP2 (biotin)	Invitrogen	Polyclonal	1.2
153	Eu	Ki67	Abcam	SP6	1.2
155	Gd	CD11b	abcam	EPR1344	1.2
156	Gd	F4/80	ab254201	D2S9R	1.2
157	Gd	CD103	Ms	EPR22590	1.5
158	Gd	CD8	Ms	D4W2Z	1
159	Tb	CD3e	Abcam	EPR22667-12	1
162	Dy	Glucagon	CST	EPR3070	1
163	Dy	Vimentin	CST	D21H3	0.75
165	Ho	Pax5	Abcam	D7H5X	1.5
166	Er	Alpha-SMA	Abcam	SP171	1
167	Er	CD19	Abcam	EPR23174	2
172	Yb	Trypsin	Abcam	EPR19498-43	2
173	Yb	SIRPα	CST	EPR22590	2
174	Yb	Beta-Tubulin	Abcam	D3U1W	3
175	Lu	CD45	Abcam	EPR20033	1
176	Lu	Insulin	Abcam	EPR173591	1

Flow markers and fluorophores

Marker	Fluorophore	Clone	Vendor	µg/10^6 cells
CD44	BUV661	IM7	BD Biosciences	0.125
CD4	BUV805	GK1.5	BD Biosciences	0.125
CD117	PE-Cy5	2B8	BioLegend	0.25
F4/80	BUV563	T45-2342	BD Biosciences	0.25
CD64	Brilliant Violet 605	X54-5/7.1	BioLegend	0.25
CD206	Brilliant Violet 711	C068C2	BioLegend	0.25
CD103	AF488	2.00E+07	BioLegend	0.25
TCRgd	PerCP-eFluor710	GL3	Invitrogen	0.25
CX3CR1	PE	SA011F11	BioLegend	0.25
Siglec-F	PE-CF594	E50-2440	BD Biosciences	0.25
CD19	BUV395	1D3	BD Biosciences	0.5
CCR2 (CD192)	BUV496	475301	BD Biosciences	0.5
CD69	BUV737	H1.2F3	BD Biosciences	0.5
Ly-6C	SuperBright 436	HK1.4	Invitrogen	0.5
CD62L	Brilliant Violet 570	MEL-14	BioLegend	0.5
CXCR2 (CD182)	Alexa Fluor 647	SA044G4	BioLegend	0.5
CD8	Alexa Fluor680	Polyclonal	Bioss	0.5
CD11b	APC-Cy7	M1/70	Tonbo Biosciences	0.5
MHC II I-Ak	BUV615	10-3.6	BD Biosciences	1
B220	Pacific Blue	RA3-6B2	BD Biosciences	1
CD11c	BV480	N418	BD Biosciences	1
CD45.1	eFluor506	A20	Invitrogen	1
CD86	BV650	GL-1	BioLegend	1
PD-L1	AF532	MIH5	Novus	1
CD66a	PerCP-Cy5.5	Mab-CC1	BioLegend	1
Ly-6G	AF594	1A8	BioLegend	1
NKp46	PE-Cy7	29A1.4	Invitrogen	1
CD3 epsilon	PerCP	145-2C11	BD Biosciences	1.5
CD16/CD32	None	93	BioLegend	0.5
Live/Dead Fixable Blue			ThermoFisher	5

T-cell proliferation reagents

Marker	Fluorophore	Clone	Vendor	Concentration
Live/Dead FB			Invitrogen	5
CFSE			Invitrogen	1
CD4	APC-Cy7	GK1.5	Fisher Scientific	0.5
Thy1.1	APC	OX7	Fisher Scientific	0.5
